# Empirically derived dietary pattern and odds of non-alcoholic fatty liver diseases in overweight and obese adults: a case–control study

**DOI:** 10.1186/s12876-022-02222-z

**Published:** 2022-03-30

**Authors:** Nasir Talenezhad, Farhang Mirzavandi, Shahab Rahimpour, Amir Pasha Amel Shahbaz, Mohammad Mohammadi, Mahdieh Hosseinzadeh

**Affiliations:** 1 Nutrition and Food Security Research Center, Shahid Sadoughi University of Medical Sciences, Yazd, Iran; 2 Department of Nutrition, School of Public Health, Shahid Sadoughi University of Medical Sciences, Yazd, Iran; 3 Gastroentrology Department, Faculty of Medicine, Shahid Sadoughi University of Medical Sciences, Yazd, Iran; 4 Department of Radiology, Faculty of Medicine, Shahid Sadoughi University of Medical Sciences, Yazd, Iran; 5 Department of Community Medicine, School of Medicine, Hormozgan University of Medical Sciences, Bandar Abbas, Iran

**Keywords:** Non-alcoholic fatty liver disease, Dietary patterns, Factor analysis, Diet

## Abstract

**Background:**

The prevalence of non-alcoholic fatty liver disease (NAFLD) is rising at an exponential rate throughout the world. Given the confirmed association between nutritional status and NAFLD, this study aimed to investigate the relationship of dietary patterns with NAFLD in overweight and obese adults.

**Methods:**

In this age- and gender-matched case–control study, 115 newly diagnosed cases and 102 control individuals participated. A validated 178-item semi-quantitative food frequency questionnaire was administered to assess the participants' dietary data. Dietary patterns were extracted from 24 predefined food groups by factor analysis. Multivariate logistic regression was run to evaluate the relationship between dietary patterns and NAFLD.

**Results:**

Factor analysis resulted in: “*western*”, “*traditional*”*,* and “*snack and sweets*” dietary patterns. The NAFLD odds were greater in participants at the highest quintile of the “western” dietary pattern than the lowest quintile (OR: 3.52; 95% CI: 1.64, 8.61). A significant increasing trend was observed in NAFLD odds across increasing quintiles of the “western” dietary pattern (P-trend = 0.01). After adjusting for the potential confounders, this relationship remained significant (OR: 3.30; 95% CI: 1.06–10.27). After full adjustments, NAFLD had no association with “traditional” or “snack and sweets” dietary patterns.

**Conclusion:**

The “western” dietary pattern containing fast food, refined grains, liquid oil, pickles, high-fat dairy, sweet desserts, red meat, tea, and coffee was associated with increased odds of NAFLD. However, further prospective studies are required to establish these results.

## Background

Non-alcoholic fatty liver disease (NAFLD), the most common chronic liver disease, is the liver symptom of metabolic syndrome and insulin resistance characterized by accumulation of triglycerides in liver cells and hepatic steatosis [1]. In developed stages (grades 3 and 4), it can lead to non-alcoholic steatohepatitis, fibrosis, cirrhosis, liver failure, and hepatocellular carcinoma [1, 2]. This disease is an independent risk factor for cardiovascular diseases [3, 4] with an increasing average prevalence of 23.71% in Europe, 5–44% in different countries, and 27% in Asia [5]. The prevalence of NAFLD is more common in the Middle East [5, 6] with a prevalence of more than 30% in the general population of Iran [7].

Nutrition is a major modifiable environmental factor in NAFLD development and management [8, 9]. Some studies investigated the association of diet with NAFLD only at the macronutrient and micronutrient levels [9–11]. For example, a review study investigated the effect of macronutrients (such as carbohydrates, fats, fructose, fiber, short-chain fatty acids, unsaturated fats, and choline) and micronutrients (such as vitamins E and C and minerals) in the development and treatment of NAFLD [9]. The findings are challenging since people consume nutrients in foods that contain a combination of nutrients; so, the effect of specific nutrients on the intended outcome is hard to interpret due to the interaction or accumulation between nutrients [12, 13].

Recently, nutritional epidemiology has adopted a more comprehensive approach, entitled “dietary pattern analysis” to examine nutritional complexities, remove previous constraints, and implement more realistic nutritional strategies at the community level [14, 15]. The results of studies on the association of dietary patterns and their components with NAFLD are limited and inconsistent [16, 17]. For instance, NAFLD had an inverse relationship with adherence to the Mediterranean [18] and Dietary Approaches to Stop Hypertension (DASH) [19] diets. Furthermore, adherence to “western” dietary patterns increased the odds of NAFLD significantly [20, 21]. However, a cross-sectional study showed no significant relationship between adherence to the “western” dietary pattern and NAFLD [22]. The results also showed that “traditional Chinese” and “high salt” diets had no association with increased risk of NAFLD [23]. Some studies found that traditional dietary pattern had no association with increased risk of general or central obesity [24] and NAFLD [25]. However, the "traditional" dietary patterns with high consumption of vegetables, fish, and mushrooms [22] as well as the "traditional Chinese" diet including whole grains, fruits, and vegetables increased and reduced the risk of fatty liver, respectively [26].

To the best of our knowledge, few inconsistent studies examined the association of dietary patterns with NAFLD and most of them were conducted in Western societies. Given the above-mentioned ideas and since dietary patterns differ among countries, especially between the Middle Eastern and Western nations and the prevalence of NAFLD is higher in these countries [5, 6], the present study was conducted. The aim was to investigate the association between dietary patterns and NAFLD odds in participants with a body mass index (BMI) of greater than 25 kg/m^2^ from October 2017 to March 2019 in Iran.

## Methods

### Study design

The present case–control research was conducted among 240 overweight and obese individuals within the age range of 20–69 years. The participants were selected from people referred to the academic liver disease clinics from October 2017 to March 2019 using the convenience sampling method. The case group (n = 120) included patients with NAFLD diagnosed based on laboratory tests and abdominal ultrasound within the previous month. The control group members (n = 120) were selected from the same clinic in the same period after matching for age and gender, but they did not have NAFLD.

The study sample size was calculated as 240 using α = 0.05 and test power of 90% [27] considering a significant odds ratio (OR) of 1.45 [28].

### Eligibility criteria

Among the participants referred to the liver clinic in Yazd, a total of 240 adults 20–69-year-old were included in our study. The participants were required to sign informed consent forms to enter the research. Inclusion criteria were individuals with a BMI of greater than 25 kg/m^2^. Individuals were excluded at the baseline in the case of (1) using drugs inducing hepatotoxicity (tamoxifen, steroids, amiodarone) and alcoholic beverages; (2) having cardiovascular diseases (coronary artery disease, congestive heart disease), diabetes type 1, chronic B or C hepatitis virus infections, cancer, Wilson's disease, hemochromatosis, biliary diseases or cirrhosis, and another liver disease; and (3) having a history of being on a special diet, such as diets of weight gain or weight loss, ketogenic, vegetarian, nordic, dietary approaches to stop hypertension (DASH), Mediterranean, Atkins, and paleo.

### Study protocol

**Fig. 1:**
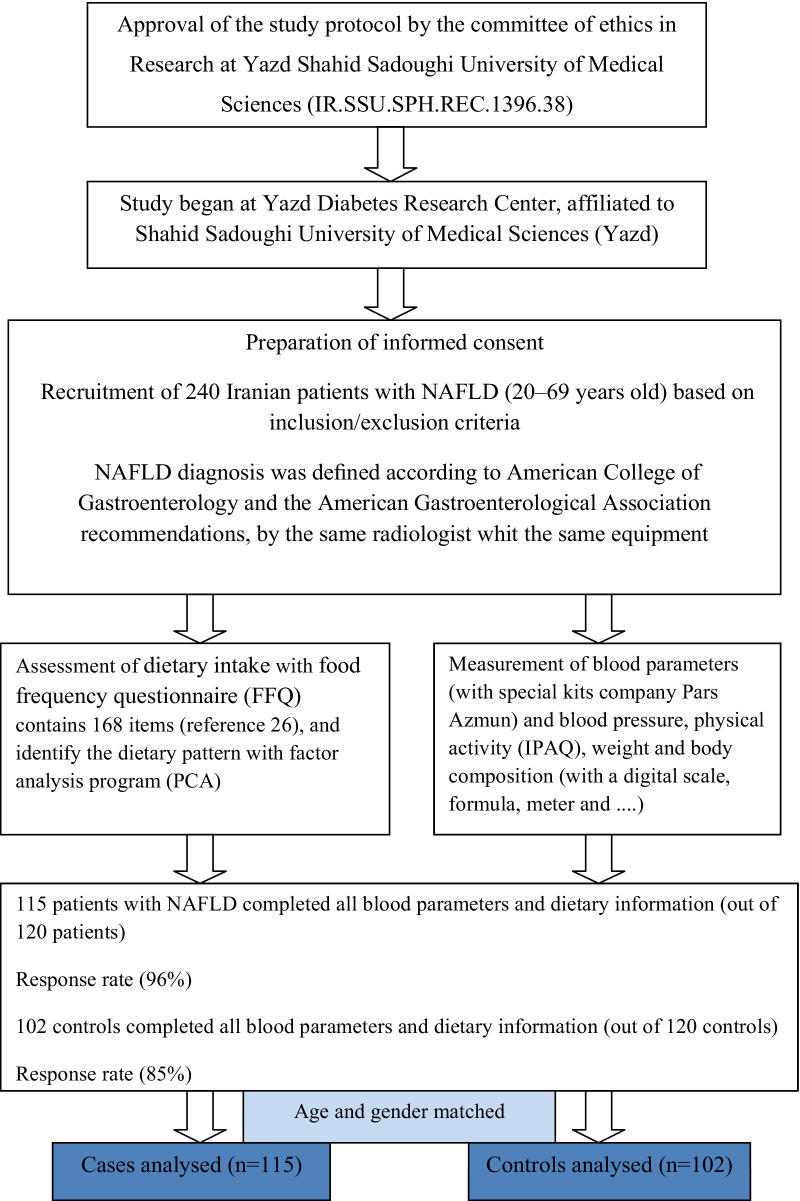
Flow chart diagram of selection and enrollment of study subjects at the present study

After signing the informed consent forms, all participants underwent an abdominal ultrasound Mindray DC-70 ultrasound machine (Mindray Building, Shenzhen, China) by the same radiologist using the same device. As a result, they were classified into two groups. The participants' recruitment procedures are represented in Fig. 1. All participants were evaluated in terms of their abdominal ultrasound and liver enzymes available in serum samples. The liver steatosis was estimated by evaluating the image brightness of the echo pattern. Abdominal ultrasound is not able to detect hepatic fat deposition in the case that it is less than 33% of the total liver weight. In this regard, individuals with a total liver weight of lower than 33% were categorized as the control group. Laboratory data (ALT, AST, and GGT) were collected from control group members and NAFLD patients after more than 12 h of fasting in enrollment. Dietary data were collected using a validated food frequency questionnaire (FFQ) [29]. Recommendations provided by the American College of Gastroenterology and the American Gastroenterological Association were also employed for the diagnosis of NAFLD [30].

### Laboratory indicators

In order to determine the serum concentrations of the hepatic enzymes as well as the glucose and lipid profiles, concentrations of liver enzymes, including Alanine aminotransferase (ALT), Aspartate Aminotransferase (AST), and Gamma-glutamyltransferase (GGT), fasting blood glucose, and lipid profiles including low-density lipoprotein cholesterol (LDL-c), high-density lipoprotein cholesterol (HDL-c), total cholesterol (TC), and triglycerides (TG) were determined. To this end, an enzymatic colorimetric assay was used in a diabetes specialist laboratory.

### Assessment of dietary intake

The participants' dietary intake was assessed during the previous year using a semi-quantitative FFQ previously validated for the Iranian population [29]. The original semi-quantitative FFQ contains 168 items, but 10 more questions were added regarding consumption of Yazd-specific frequently consumed food items, which made a total of 178 items. Trained interviewers completed questionnaires after asking the participants to report the amount and frequency of each food item consumption daily (once to four times, five to seven times, seven to nine times, 10 times and more), weekly (once, two to four times, five to six times), and monthly (never or less than once, one to three times) in the past year. Participants were also asked about their usual consumption rate of each food item. A photo book was applied as a reference so that participants could estimate the portion size of foods accurately. Food supplements were also assessed by FFQ. Daily nutrient and energy intakes of each participant were calculated using Nutritionist IV software based on the US Department of Agriculture’s national nutrient databank. People with an energy intake of less than 800 kcal/day or greater than 4000 kcal/day were excluded from the study (n = 23). Food intake information was converted into grams per day for final analysis. To identify dietary patterns, food items were assigned into 24 predefined food groups included in factor analysis.

### Anthropometric and physical activity measurements

The participants' weight, fat mass, muscle mass, and visceral fat were measured in light clothes with no shoes by a digital scale (Omron Digital Scale, Model BF511) with 5 g precision by a trained nutritionist. Later, BMI was calculated after dividing weight in kilograms by height in meters squared. Participants' height was measured using a tape meter with 5 cm precision in a standing position without shoes. Waist circumference (WC) was measured in the thinnest area between the ribs and the iliac crest. Hip circumference (HC) measurements were performed by a non-elastic tape meter without any pressure on the body. The International Physical Activity Questionnaire (IPAQ) was used to assess physical activity [31].

### Blood pressure measurements

On the visit day, the participant's blood pressure was assessed by a digital pressure indicator (Citizen Japan Company, CH456 model) after fasting for the past hour, without any intense physical activity, and 10 min of rest on the chair.

### Assessment of other variables

Data on participants' age, gender, education (High school, Diploma, Associate Degree, Bachelor's and higher), job (Housewife, Employee, Free job), history of diabetes (No, Yes), tobacco and alcohol (No, Yes: used as an exclusion criterion), as well as medications and dietary supplements (No, Yes) were obtained.

### Ethical considerations

The study protocol was approved by the Ethics Committee in Yazd Shahid Sadoughi University of Medical Sciences (IR.SSU.SPH.REC.1396.38). Furthermore, written informed consents were obtained from all participants.

### Statistical analysis

All data were entered into SPSS for statistical analysis. Independent t-test and chi-square test were run to compare continuous and categorical variables between the two groups, respectively. The factor analysis was applied to determine dietary patterns. To this end, the study factors were naturally interpreted in conjunction with eigenvalues > 1.5 and the scree plot were depicted. The derived dietary patterns were labeled according to data interpretation and similar studies. To calculate the factor score of each pattern, the food group intakes weighted by their factor loadings were summed for each participant. Varimax rotation was selected to create a simple and differentiated matrix; later, the most correlated items were introduced as a pattern. A score was calculated for each individual in each pattern. These scores were used as independent variables in subsequent analyses to identify the association of dietary patterns with dependent variables. Moreover, analysis of variance was performed to compare quantitative variables between quintiles of each dietary pattern. A multiple logistic regression model was applied based on the ORs and the 95% confidence intervals to examine the relationship between fatty liver and dietary pattern quartiles in crude and adjusted models. *P* values less than 0.05 were considered statistically significant.

## Results

### Participants' characteristics

A total of 217 participants were investigated in the case (n = 115) and control (n = 102) groups. The mean age of the case and control groups were 44.22 ± 10.35 and 43.52 ± 12.14 years, respectively. Table 1 shows the participants' demographic, biochemical, and anthropometric characteristics.

**Table 1:** General characteristics, energy and physical activity

Variables	Case (n = 115)	Control (n = 102)	*P* value^a^
Age (year)	44.22 ± 10.35	43.52 ± 12.14	0.64
Sex			
Female	65	57	0.92
Male	50	45	
Weight (kg)	84.6 ± 16.34	68.98 ± 11.57	0.00
Height	160.61 ± 24.57	163.02 ± 18.31	0.42
BMI (kg/m^2^)	30.39 ± 4.10	25.26 ± 3.94	0.00
WC (cm)	102.78 ± 12.62	93.36 ± 9.78	0.00
HC (cm)	111.13 ± 9.77	102.40 ± 8.34	0.00
Fat mass	37.60 ± 10.22	31 ± 9.8	0.00
Muscle mass	27.89 ± 5.58	29.59 ± 6.27	0.03
Visceral fat	11.51 ± 3.46	7.12 ± 2.84	0.00
Blood pressure (BP)			
Systolic BP	129.28 ± 19.16	115.99 ± 13.30	0.00
Diastolic BP	74.89 ± 9.35	71.47 ± 9.25	0.00
FBS	120.77 ± 47.74	107.14 ± 26.07	0.01
TG	200.51 ± 89.62	141.07 ± 69	0.00
TC	185.21 ± 46.73	165.42 ± 44.86	0.32
LDL-c	114.59 ± 35.77	103.44 ± 34.83	0.02
HDL-c	42.08 ± 9.34	45.73 ± 18.07	0.06
AST	28.59 ± 14.90	17.58 ± 5.51	0.00
ALT	42.13 ± 34.78	19.75 ± 8.61	0.00
Job			
Housewife	59	40	0.20
Employee	30	33	
Self-employment	26	29	
Education			
High school	44	34	
Diploma	33	22	0.31
Associate degree	5	5	
Bachelor's and higher	33	41	
Diabetes			
No	76	26	0.22
Yes	77	38	
PA (MET-min/week)			
< 1 h	32	70	
> 1 h	48	67	0.11
Energy intake (Kcal)	2274.08 ± 670.25	2050.12 ± 722.69	0.01

*BMI* body mass index, *WC* waist circumference, *HC* hip circumference, *FBS* fasting blood sugar, *TG* triglyceride, *TC* total cholesterol, *AST* aspartate aminotransferase, *ALT* alanine aminotransferase, *PA* physical activity

^a^*P* values resulted from independent t tests for quantitative and Chi-square for qualitative variables between the two groups

Patients with NAFLD had higher weight, systolic and diastolic blood pressure, and more energy intake than the control group. Serum levels of FBS (*P* = 0.01), TG (*P* < 0.001), LDL-c (*P* = 0.02), AST (*P* < 0.001), ALT (*P* < 0.001) were higher in participants with NAFLD than the controls. No significant difference was found in means of TC and HDL-c between the two groups (*P* > 0.05).

To analyze the dietary patterns, 178 food items available in FFQ were categorized under 24 food groups (Table 2). As a result, major dietary patterns of western (fast food, refined grains, liquid oil, pickles, high-fat dairy, sweet desserts, red meat, tea, and coffee), traditional (vegetables, cereals, fruits, organ meats, low-fat dairy, poultry, and nuts), and snack/sweets (soft drinks, snacks, sweet desserts, sugars, and nuts) were determined. Three dietary patterns explained 26.65% of the total variance in dietary intakes. Factor-loading matrixes for these dietary patterns were explained in Table 3.

**Table 2:** Food groups used in the analyses of dietary pattern

Food groups	Food items
Processed meats	Sausages
Red meats	Lamb meat, veal meat, minced meat
Organ meats	Lamb(liver, kidneys, heart, tongue, brain, stomach, kidney, foot)
Fish	Fish, tuna
Poultry	Chicken with skin, chicken without skin
Eggs	Eggs
Low fat dairy product and milk	Dough, low fat yoghurt, low fat milk, cheese, high fat milk,, chocolate milk, flavored Milk
High fat dairy product	High fat yogurt, ordinary yogurt, creamy yogurt, cream cheese, cream, Industrial and traditional ice cream, curd
Tea coffee	Tea, coffee
Fruit	Apples, cherries, apricots, plums, fresh figs, dry figs, kiwi, strawberries, grapes, fresh berries, dry berries, dates, barberries, bananas, pomegranates, Peach, nylon, cantaloupe, melon, pear, nectarine, green tomato, grapefruit, orange, persimmon, tangerine, cherry, sweet lemon, sour lemon, watermelon, raisin, fresh pineapple, dried peach and apricot
Fruit juice	Grapefruit juice, orange juice, apple juice, cantaloupe juice
Other vegetables	Pumpkin, zucchini, green cucumber, eggplant, celery, green peas, green beans, okra, raw onions, turnips, beets, cooked mushrooms, corn, fresh vegetables, stewed vegetables, raw and cooked carrots, cabbage ketchup, tomato sauce, tomato, spinach, lettuce, garlic, cooked potatoes, black pepper, fried onions
Fast food	hamburger, fried potatoes, pizza
Whole grain	Sangak, Taftoon, Corno, oat bread,biscuit with bran
Refined grain	Bread (Lavash, Baguette, Barbari, Toast, dried), rice, flour, barley, noodles and vermicelli, macaroni, biscuit
snacks	Chips and puffs
Nuts	Peanuts, almonds, walnuts, pistachios, hazelnuts, seeds
Vegetables oil	Liquid oil, olive oil, olives
Sweete dessert	Noghl, pirashki, qotab, baqlava, loz, pashmak, hajibadam1, nan_berenji, Sohan (Iranian sweets), chocolates, caramel cream, cookies, Fresh sweets, dry sweets, halva, arde, halva arde, Yazdi Cake, cakes, candy, Jelly, honey, jam, lemon juice, canned pineapple, compote fruits, lemon juice
Hydrogenated fats	Animal fat, solid oil, mayonaise, Fat, cream, butter, margarine, broth
Sugars	Sugar, ghand, nabat, gaz (Iranian sweet)
Soft drink	Soft drinks
Pickles	Salinity cucumber, mixed vegetable pickles, salt
Legumes	green peas, lentils, beans, chickpeas, soybeans, split peasو broad bean, mung bean, cotyledon

**Table 3:** Rotated factor loading matrix for the major dietary patterns

Food group	Western dietary pattern	Traditional dietary pattern	Snack and sugar dietary pattern
Fast food	0.728	–	–
Refined grains	0.587	–	–
Liquid oil	0.526	–	–
Pickles	0.525	–	–
High-fat dairy	0.480	–	–
Solid oil	− 0.375	–	–
Red Meat	0.347	0.301	–
Tea and coffee	0.311	–	–
fishes	–	–	–
Fruit juices	–	–	–
Other vegetables	–	0.714	–
legumes	–	0.611	–
Fruits	–	0.553	–
Organ meats	–	0.478	–
Low-fat dairy	–	0.475	–
Poultry	–	0.456	–
Egg	–	–	–
Soft drinks	–	–	0.726
Snack	–	–	0.629
Nuts	–	0.418	0.526
Sweet desserts	0.420	–	0.496
Sugar	–	–	0.389
Whole grains	0.320	–	− 0.367
Processed meats	–	–	–
Percentage of variance			
Explained^a^	9.96	8.71	7.91

Only items with correlation coefficients ≥ [0·30] were presented

^a^Cumulative percentage of variance explained by three dietary patterns was 26.58%

Table 4 contains the participants’ characteristics in quintiles of the dietary patterns. In the “western” dietary pattern, the highest quintile included males, higher education levels, employee and self-employment occupations, without physical activity, higher prevalence of diabetes, and BMI ≥ 25 kg/m^2^. Conversely, participants in the lowest quintile of the “traditional” dietary pattern were female housekeepers without the prevalence of diabetes and BMI ≤ 25 kg/m^2^. Participants in the lowest quintile of the “snack and sugar” dietary pattern were female housekeepers with BMI ≤ 25 kg/m^2^, high school education level, and prevalence of diabetes.

**Table 4:** Distribution of general characteristics of the study subjects in different categories of dietary patterns

Variable	Western Dietary pattern				Traditional Dietary pattern				Snack and sugar Dietary pattern			
	Q1 (n = 43)	Q3 (n = 43	Q5 (n = 43)	*P*^a^	Q1 (n = 43)	Q3 (n = 43)	Q5 (n = 43)	*P*^a^	Q1 (n = 43)	Q3 (n = 43)	Q5 (n = 43)	*P*^a^
	N (%)	N (%)	N (%)		N (%)	N (%)	N (%)		N (%)	N (%)	N (%)	
Sex												
Female	28 (65.1%)	23 (53.5%)	21 (48.8%)	0.61	26 (60.5%)	21 (48.8%)	20 (46.5%)	0.35	29 (67.4%)	23 (53.5%)	22 (51.2%)	0.46
Male	15 (34.9%)	20 (45.5%)	22 (51.2%)		17 (39.5%)	22 (51.2%)	23 (53.5%)		14 (32.6%)	20 (46.5%)	21 (48.8%)	
Job												0.15
Housewife	21 (48.8%)	19 (44.2%)	17 (39.5%)	0.67	20 (46.5%)	18 (41.9%)	14 (32.6%)	0.34	27 (69.8%)	18 (41.9%)	19 (44.2%)	
Employee	12 (27.9%)	17 (39.5%)	13 (30.2%)		14 (32.6%)	11 (25.6%)	13 (30.2%)		6 (14%)	17 (39.9%)	10 (23.3%)	
Self-employment	10 (23.3%)	7 (16.3%)	13 (30.2%)		9 (20.9%)	14 (32.6%)	16 (37.2%)		10 (23.3%)	8 (18.6%)	14 (32.6%)	
Education												
High school	22 (51.2%)	12 (27.9%)	10 (23.3%)		14 (32.6%)	17 (39.5%)	15 (34.9%)	0.88	25 (58.1%)	10 (23.3%)	12 (27.9%)	0.07
Diploma	8 (18.6%)	10 (23.3%)	11 (25.6%)	0.05	13 (30.2%)	8 (18.6%)	15 (34.9%)		10 (23.3%)	12 (27.9%)	13 (30.2%)	
Associate degree	3 (7%)	1 (2.3%)	5 (11.6%)		3 (7%)	1 (2.3%)	2 (4.7%)		2 (4.7%)	4 (9.3%)	1 (2.3%)	
Bachelor's and higher	10 (23.3%)	20 (46.5%)	17 (39.5%)		13 (30.2%)	17 (39.5%)	11 (25.6%)		6 (14%)	17 (39.5%)	17 (39.5%)	
Diabetes												
No	26 (60.5%)	32 (74.4%)	35 (81.4%)	0.13	32 (74.4%)	29 (67.4%)	28 (65.1%)	0.85	18 (41.9%)	37 (86%)	32 (74.4%)	0.00
Yes	17 (39.5%)	11 (25.6%)	8 (18.6%)		11 (25.6%)	14 (32.6%)	15 (34.9%)		25 (58.1%)	6 (14%)	11 (25.6%)	
PA (MET-min/week)				0.70				0.91				0.00
< 1 h	15 (34.9%)	14 (32.6%)	19 (44.2%)		16 (37.2%)	16 (37.2%)	18 (41.9%)		22 (51.2%)	13 (30.2%)	24 (55.8%)	
> 1 h	28 (65.1%)	29 (67.4%)	24 (55.8%)		27 (62.8%)	27 (62.8%)	25 (58.1%)		21 (48.8%)	30 (69.8%)	19 (44.2%)	
BMI (kg/m^2^)												
< 25	23 (53.5%)	15 (34.9%)	10 (23.3%)	0.01	17 (39.5%)	9 (20.9%)	19 (23.3%)	0.07	15 (34.9%)	12 (27.9%)	12 (27.9%)	0.86
> 25	20 (46.5%)	28 (65.1%)	33 (76.7%)		26 (60.5%)	34 (79.1%)	33 (76.7%)		28 (65.1%)	31 (72.1%)	31 (72.1%)	

*BMI* body mass index, *PA* physical activity

^a^ANOVA for continuous variables and χ^2^ test for categorical variables were used. For quantitative variables mean ± SD; and for qualitative variables frequency (percentage) were used

The participants' anthropometric indices across different dietary patterns are shown in Table 5. Regarding the “western” dietary pattern, individuals in the lowest quintiles were older with significantly lower weight, BMI, WC, HC, fat mass, muscle mass, and visceral fat. Participants in higher quintiles of the “traditional” dietary pattern had higher BMI, WC, and visceral fat compared. Participants in the lowest quartile of the “snack and sugar” dietary pattern were younger.

**Table 5:** Characteristics of anthropometric indices of subjects in different categories of dietary patterns

Variable	Western dietary pattern				Traditional dietary pattern				Snack and sugar dietary pattern			
	Q1 (mean ± SE)	Q3 (mean ± SE)	Q5 (mean ± SE)	*P*^a^	Q1 (mean ± SE)	Q3 (mean ± SE)	Q5 (mean ± SE)	*P*^a^	Q1 (mean ± SE)	Q3 (mean ± SE)	Q5 (mean ± SE)	*P*^a^
Age (year)	48.07 ± 2.03	44.00 ± 1.64	40.86 ± 1.36	0.02	42.62 ± 1.90	42.62 ± 1.90	42.69 ± 1.49	0.17	49.97 ± 1.83	41.60 ± 1.58	42.62 ± 1.48	0.00
Weight (kg)	69.62 ± 2.03	74.38 ± 1.88	83.23 ± 2.52	0.00	74.93 ± 2.37	79.96 ± 2	82.75 ± 2.51	0.01	74.27 ± 2.04	76.52 ± 2.10	83.95 ± 3.45	0.04
Height (cm)	162.47 ± 1.23	160.52 ± 4.08	165.90 ± 1.44	0.65	159.24 ± 4.20	159.24 ± 4.20	163.06 ± 4.17	0.82	161.06 ± 1.79	163.57 ± 1.47	160.64 ± 4.26	0.97
BMI (kg/m^2^)	25.98 ± 0.74	27.52 ± 0.74	29.03 ± 0.74	0.01	27.05 ± 0.81	27.05 ± 0.81	28.64 ± 0.67	0.02	28.19 ± 0.78	27.93 ± 0.61	29.05 ± 0.82	0.12
WC (cm)	91.81 ± 2.49	98.24 ± 1.61	100.66 ± 1.31	0.00	97.80 ± 2.13	97.80 ± 2.13	100.54 ± 1.56	0.00	98.37 ± 1.78	98.31 ± 1.28	102.30 ± 1.60	0.20
HC (cm)	102.11 ± 1.37	106.26 ± 1.59	109.28 ± 1.69	0.00	105.92 ± 1.68	105.92 ± 1.68	108.83 ± 1.72	0.27	106.88 ± 1.46	105.73 ± 1.02	109.97 ± 1.93	0.37
Fat mass (%)	33.62 ± 1.68	32.41 ± 1.65	34.29 ± 1.61	0.39	34.05 ± 1.84	34.05 ± 1.84	34.34 ± 1.81	0.97	36.28 ± 1.68	33.36 ± 1.19	35.05 ± 1.67	0.52
Muscle mass (%)	28.14 ± 0.87	30.00 ± 1.03	28.87 ± 0.86	0.57	27.92 ± 0.97	27.92 ± 0.97	29.52 ± 0.98	0.80	27.55 ± 0.84	28.92 ± 0.75	29.13 ± 0.91	0.35
Visceral fat (%)	8.08 ± 0.52	9.58 ± 0.63	10.33 ± 0.69	0.12	8.47 ± 0.59	8.47 ± 0.59	10.95 ± 0.70	0.00	9.48 ± 0.56	9.42 ± 0.72	10.03 ± 0.65	0.52

*BMI* body mass index, *WC* waist circumference, *HC* hip circumference

^a^ANOVA for continuous variables and χ^2^ test for categorical variables were used. For quantitative variables mean ± SD; and for qualitative variables frequency (percentage) were used

Table 6 contains the average intake of food groups in different categories of dietary patterns. In “western” dietary pattern, a significant difference was observed regarding intake of fast food, refined grains, liquid oil, pickles, high-fat dairy, red meat, tea and coffee, fish, other vegetables, legumes, fruits, low-fat dairy, snacks, sweet desserts, sugar, whole grains, and processed meats in high quintiles (*P* < 0.05). However, average consumption rates of poultry, egg, fruit juices, solid oils, soft drinks, organ meats, and nuts were not significant (*P* > 0.05). The mean intake of refined grains, liquid oil, red meat, tea and coffee, other vegetables, legumes, fruits, organ meats, low-fat dairy, poultry, nuts, and whole grains was significantly different among the “traditional” dietary pattern quintiles (*P* < 0.05). Participants with higher adherence to the “snack and sugars” dietary pattern had a higher intake of refined grains, high-fat dairy, legumes, soft drinks, snacks, nuts, sweet desserts, sugar, whole grains, processed meats, and fast food (*P* < 0.05).

**Table 6:** The average consumption of food groups and nutrients in different categories of dietary patterns explored

Food groups (g)	Western dietary pattern				Traditional dietary pattern				Snack and sugar dietary pattern			
	Q1 (mean ± SE)	Q3 (mean ± SE)	Q5 (mean ± SE)	*P*^a^	Q1 (mean ± SE)	Q3 (mean ± SE)	Q5 (mean ± SE)	*P*^a^	Q1 (mean ± SE)	Q3 (mean ± SE)	Q5 (mean ± SE)	*P*^a^
Fast food	7.72 ± 0.88	16.90 ± 1.99	37.56 ± 5.19	0.00	24.39 ± 4.38	20.93 ± 3.67	22.79 ± 5.25	0.36	10.62 ± 1.65	17.78 ± 2.94	38.79 ± 6.43	0.00
Refined grains	101.48 ± 8.51	257.26 ± 16.94	372.50 ± 25.80	0.00	210.37 ± 18.88	287.79 ± 28.64	236.25 ± 16.78	0.00	219.94 ± 25.88	181.79 ± 15.70	280.63 ± 22.55	0.01
Liquid oil	4.62 ± 0.62	7.83 ± 1.08	8.45 ± 0.91	0.01	4.49 ± 0.56	7.60 ± 0.93	7.36 ± 0.71	0.01	5.68 ± 0.64	7.29 ± 1.06	7.65 ± 0.78	0.41
Pickles	8.57 ± 1.36	14.70 ± 1.96	28.32 ± 3.89	0.00	13.23 ± 2.19	16.62 ± 2.54	18.87 ± 2.70	0.13	15.53 ± 2.98	18.68 ± 3.10	23.09 ± 3.00	0.10
High-fat dairy	26.49 ± 2.55	36.75 ± 4.72	78.56 ± 9.70	0.00	46.20 ± 6.19	50.65 ± 6.92	60.77 ± 7.74	0.13	40.78 ± 7.34	44.03 ± 6.11	67.69 ± 8.74	0.02
Solid oil	69.82 ± 13.05	43.36 ± 6.40	44.32 ± 4.96	0.14	42.93 ± 6.41	67.26 ± 12.32	53.19 ± 5.95	0.23	60.66 ± 11.45	42.60 ± 5.15	50.79 ± 6.72	0.58
Red Meat	35.59 ± 4.64	48.79 ± 5.14	66.71 ± 8.05	0.00	38.77 ± 4.70	53.35 ± 6.74	57.39 ± 6.82	0.01	41.09 ± 5.67	49.18 ± 6.56	63.31 ± 6.86	0.13
Tea and coffee	157.89 ± 16.37	292.30 ± 27.12	374.72 ± 38.87	0.00	214.68 ± 27.59	277.32 ± 21.60	269.96 ± 31.88	0.02	251.30 ± 26.97	290.50 ± 29.29	271.50 ± 26.84	0.62
fishes	6.12 ± 0.83	11.16 ± 1.35	17.62 ± 3.14	0.00	8.99 ± 1.57	15.07 ± 5.95	16.44 ± 2.26	0.43	7.98 ± 1.19	12.31 ± 2.24	15.57 ± 2.31	0.23
Fruit juices	47.22 ± 10.67	33.51 ± 5.35	57.67 ± 7.94	0.11	36.40 ± 6.34	38.05 ± 5.67	64.91 ± 10.24	0.08	32.56 ± 5.19	57.05 ± 9.93	49.64 ± 8.14	0.10
Other vegetables	216.57 ± 16.36	314.75 ± 24.06	499.06 ± 42.02	0.00	169.20 ± 11.88	319.04 ± 15.91	538.48 ± 43.20	0.00	311.01 ± 27.47	307.34 ± 22.39	382.58 ± 43.69	0.11
legumes	22.65 ± 2.13	29.77 ± 3.34	42.99 ± 3.96	0.00	21.27 ± 1.55	33.13 ± 2.78	45.11 ± 4.99	0.00	40.71 ± 4.15	33.64 ± 3.73	27.31 ± 2.63	0.02
Fruits	526.41 ± 57.90	749.81 ± 66.60	781.73 ± 59.24	0.00	242.73 ± 21.19	582.37 ± 24.14	1239.16 ± 61.52	0.00	611.71 ± 64.43	651.94 ± 65.73	674.40 ± 63.01	0.78
Organ meats	6.59 ± 0.73	6.11 ± 0.55	8.72 ± 1.16	0.14	5.79 ± 0.56	6.04 ± 0.49	9.62 ± 1.19	0.00	5.86 ± 0.46	6.17 ± 0.55	7.96 ± 0.95	0.27
Low-fat dairy	107.24 ± 10.71	167.30 ± 16.16	351.72 ± 39.95	0.00	98.05 ± 8.89	229.53 ± 21.68	317.38 ± 39.34	0.00	208.93 ± 27.56	200.47 ± 33.61	204.41 ± 20.22	0.88
Poultry	45.51 ± 7.07	59.20 ± 6.65	72.43 ± 9.05	0.34	38.50 ± 4.99	57.32 ± 9.01	76.88 ± 10.95	0.01	52.03 ± 7.51	72.99 ± 12.53	65.77 ± 8.62	0.18
Egg	22.60 ± 3.48	28.01 ± 4.05	32.56 ± 2.90	0.19	25.93 ± 3.91	32.36 ± 3.03	29.51 ± 3.63	0.46	30.94 ± 4.29	28.83 ± 3.46	35.44 ± 4.18	0.34
Soft drinks	27.40 ± 5.46	66.57 ± 17.35	58.75 ± 12.15	0.07	71.87 ± 18.25	82.62 ± 17.40	45.66 ± 10.38	0.12	11.72 ± 1.09	26.49 ± 2.28	195.00 ± 20.17	0.00
Snack	3.30 ± 0.61	8.71 ± 2.45	6.97 ± 1.35	0.04	6.39 ± 1.74	5.53 ± 0.92	7.62 ± 2.41	0.14	3.24 ± 0.33	3.97 ± 0.46	13.99 ± 2.84	0.00
Nuts	8.66 ± 1.42	16.85 ± 2.95	20.01 ± 2.93	0.13	10.69 ± 2.15	12.28 ± 1.84	22.04 ± 3.24	0.00	8.12 ± 1.25	13.18 ± 1.92	22.76 ± 3.59	0.00
Sweet desserts	39.68 ± 4.42	58.80 ± 5.16	88.57 ± 11.22	0.00	65.86 ± 9.96	58.15 ± 4.97	77.28 ± 9.56	0.07	34.36 ± 2.86	55.86 ± 3.49	120.069 ± 12.24	0.00
Sugar	11.37 ± 1.98	17.09 ± 2.02	14.52 ± 2.02	0.02	20.81 ± 3.37	15.97 ± 2.31	16.46 ± 1.89	0.25	10.10 ± 2.05	19.90 ± 2.55	22.82 ± 3.12	0.00
Whole grains	32.63 ± 3.40	46.10 ± 5.62	58.89 ± 6.29	0.02	33.54 ± 4.14	41.92 ± 4.11	50.58 ± 5.97	0.01	73.63 ± 7.92	35.10 ± 3.69	36.85 ± 3.51	0.00
Processed meats	2.32 ± 0.36	3.52 ± 1.03	3.99 ± 0.83	0.03	8.22 ± 2.59	2.97 ± 0.64	4.05 ± 0.95	0.10	1.90 ± 0.17	3.32 ± 0.64	8.25 ± 1.65	0.00

^a^ANOVA for continuous variables and χ^2^ test for categorical variables were used. For quantitative variables mean ± SE

### Dietary patterns and NAFLD

The associations of dietary patterns with NAFLD risk are shown in Table 7. In the crude model, the risk of NAFLD was 3.52 times higher in participants at the top quintile of the “western” dietary pattern (OR: 3.52; 95% CI: 1.64, 8.61). A significant increasing trend was observed in NAFLD odds across higher quintiles of the western dietary pattern (P-trend = 0.01). Adjustment for energy intake, education, Job, diabetes disease history, medication and supplements, and physical activity in model 2 indicated significant odds of NAFLD from the fourth quintile (OR: 3.30; 95% CI: 1.06–10.27). In the crude model, a higher score for the "traditional” and “snack and sugar” dietary patterns were not associated with increased NAFLD odds (OR: 2.35; 95% CI: 0.98, 5.62) and (OR: 0.91; 95% CI: 0.38, 2.21), respectively. After adjusting for potential confounders, higher adherence to “traditional” and “Snack and sugar” dietary patterns was not associated with NAFLD odds.

**Table 7:** Results of a logistic regression model to investigate the relationship between Nonalcoholic fatty liver disease and dietary pattern quintiles

Dietary pattern	Q1	Q2	Q3	Q4	Q5	*P*^c^
	OR (CI 95%)	OR (CI 95%)	OR (CI 95%)	OR (CI 95%)	OR (CI 95%)	
Western						
Crude	1	3.66 (1.50–8.92)	2.65 (1.09–6.24)	3.33 (1.37–8.08)	3.52 (1.64–8.61)	0.01
Model 1^a^	1	3.31 (1.30–8.42)	2.29 (0.86–6.08)	2.72 (0.95–7.78)	2.64 (0.79–8.83)	0.28
Model 2^b^	1	4.43 (1.60–12.26)	3.12 (1.07–9.09)	3.30 (1.06–10.27)	3.76 (0.97–14.48)	0.19
Traditional						
Crude	1	0.79 (0.33–1.87)	1.75 (0.74–4.12)	1.82 (0.77–4.27)	2.35 (0.98–5.62)	0.01
Model 1^a^	1	0.79 (0.33–1.86)	1.59 (0.65–3.89)	1.54 (0.58–4.04)	1.90 (0.68–5.39)	0.14
Model 2^b^	1	0.69 (0.76–1.73)	1.54 (0.59–4.04)	1.10 (0.59–3.19)	1.43 (0.46–4.42)	0.45
Snack and sugar						
Crude	1	1.04 (0.44–2.43)	0.82 (0.35–1.93)	0.72 (0.31–1.68)	0.91 (0.38–2.21)	0.56
Model 1^a^	1	0.99 (0.41–2.35)	0.78 (0/32–1.85)	0.57 (0.23–1.37)	0.57 (0.22–1.46)	0.12
Model 2^b^	1	1.22 (0.47–3.14)	0.93 (0.35–2.48)	0.80 (0.30–2.14)	0.77 (0.28–2.12)	0.21

^a^Adjusted for energy intake (Kcal/day)

^b^Adjusted for energy intake (Kcal/day), Education (high school, diploma, associate degree, bachelor's and higher), Job (housewife, employee, and free job), Diabetes disease history (yes/no), consumption of medication and supplements (yes/no), Physical activity (< 1 h, > 1 h). OR, Odds Ratio; CI, Confidence Interval

^c^*P* value trend

## Discussion

The present case–control study was conducted for the first time to investigate the relationship between dietary patterns and NAFLD risk in Yazd City, Iran. According to the findings, higher adherence to the “western” dietary pattern increased the odds of NAFLD, which is in the same line with some previous studies. In adolescents, adherence to a “western” dietary pattern including high amounts of refined grains, red meat, processed meat, seafood, dairy products, carbonated beverages, alcoholic beverages, and coffee increased the risk of NAFLD [26]. A cross-sectional study of 995 people in Australia showed that adherence to a "western" dietary pattern containing carbonated beverages, high-fat dairy, refined grains, red meat, processed meat, fried potatoes, cakes, and biscuits increased the risk of NAFLD. Obesity and overweight play the mediating role in increasing the risk of NAFLD in “western” dietary patterns [21]. To address this problem, our results were adjusted also for weight and body composition [21]. The “western” pattern contains saturated and trans fatty acids as well as high-fructose sources such as sweetened drinks and desserts, which eventually lead to the production and accumulation of fats in the liver and increase the risk of NAFLD [32–34]. In other words, some components of this dietary pattern lead to NAFLD by supplying additional energy and large amounts of sugar, such as fructose [35]. Refined grains, a component of the “western” dietary pattern, not only increase the risk of hepatic steatosis but also cause insulin resistance along with other components of this pattern, such as foods with a high glycemic index [36, 37]. Insulin resistance increases the risk of obesity and fatty liver through de novo lipogenesis [37, 38]. In this dietary pattern, consumption of vegetable oils, as important sources of omega-3 and omega-6 fatty acids may cause favorable effects on the prevalence of NAFLD in patients [39, 40]. However, in the "western" dietary patterns, the effect of vegetable oils on NAFLD recovery may be neutralized by consumption of sugary drinks, refined grains, and fast foods.

We found that adherence to a "traditional" food pattern including red meat, vegetables, beans, fruits, fruits, low-fat dairy, poultry, nuts, and sheep's head*,* trotters, and viscera had no significant association with the odds of developing fatty liver. A study over the risk of developing NAFLD among 999 Chinese adults showed that “traditional Chinese” and “high salt” dietary patterns had no association with increased risk of NAFLD, which confirms our findings [23]. Other studies also found no association between adherence to "traditional" dietary patterns and increased risk of general or central obesity [24] and NAFLD [25]. Based on a study, no significant relationship was found between adherence to this dietary pattern and changes in liver enzyme levels [41]. Contrary to our results, a cross-sectional study showed that adherence to the “traditional” dietary pattern containing high intakes of vegetables, fish, mushrooms, fermented soybeans, and seaweed increased the risk of NAFLD [22]. Moreover, a “traditional” Chinese diet including whole grains, fruits, and vegetables reduced the risk of fatty liver [26]. Although our “traditional” diet was rich in protective components, such as nuts [42], vegetables [42, 43], as well as fruits and beans [43], consumption of viscera and meat is high in this pattern, which can increase the risk of fatty liver by increasing the inflammatory cytokines and decreasing the anti-inflammatory factors [44]. In addition, most foods contained in this pattern have low fat and high carbohydrates; so, their consumption can increase the risk of NAFLD [45]. Some pieces of evidence indicate that dietary intake of the traditional dietary pattern differs between men and women; this can justify the differences in individuals' responses and the effects of this pattern on the risk of NAFLD in these individuals [46]. Inconsistency of the results can partly refer to the variety in traditional Iranian dietary pattern components and adjusted potential confounders such as total energy and physical activity.

Our results showed that the “snack and sweets” dietary patterns, including sweetened drinks, snacks, and nuts had no association with the odds of developing fatty liver after full adjustments. In accordance with our results, intake of high-calorie snacks with meals does not lead to fat accumulation in the liver [47]. Moreover, snacks with high-glycemic-load carbohydrates were directly associated with NAFLD [48]. One study found that higher consumption of non-alcoholic beverages was associated with an increased risk of NAFLD [28]. A meta-analysis of six cross-sectional studies confirmed these results [49]. However, the dietary pattern in our study also contained nuts, which improve liver cell activity and reduce the risk of NAFLD since they contain unsaturated fatty acids [50, 51]. As a result, after adjusting for the confounders such as total energy intake, weight and body composition, the interaction between components of this dietary pattern had no association with the odds of NAFLD.

Some strengths of this study include the following issues: We examined dietary patterns containing all foods and nutrients consumed in the studied population; in other words, our research was not limited to specific food items or nutrients [52]. The questionnaires were completed by a trained interviewer blinded to the participants' categorization in the case or control group, which minimized the reporting error. We used newly diagnosed individuals with NAFLD (Incident case) as a case group. Various confounders associated with fatty liver and also dietary patterns were adjusted, particularly the total energy intake as well as body fat and muscle percentage. This study also has some limitations. Lack of measurement the non-invasive markers of fibrosis such as FIB-4 or liver stiffness measurement (LSM). As a convenience sampling method has been used for selecting the patients of this study and no consecutive patients were considered, it should be kept in mind that there might be selection biases impacting the analysis. Although the FFQ is a valid tool in nutritional epidemiology [53], it may also generate random and systematic errors [16, 17]. Factor analysis could include several personal decisions, including food items' grouping or deciding on the number of patterns in extracting and naming factors. Some points should also be considered in interpreting the results: Due to the case–control design of the study, determining a clear causal relationship was impossible between dietary pattern adherence and NAFLD. Since the case group members included newly diagnosed NAFLD patients, the probability of change in their dietary patterns was low after the disease diagnosis. In addition to controlling for the potential confounders in the present study, the effects of immeasurable residual confounding variables should be considered.

## Conclusions

Based on the results, higher adherence to the “western” dietary pattern was significantly associated with higher odds of NAFLD. No significant association was observed between adherence to “snack and sweets” and “traditional” dietary patterns and the risk of developing NAFLD. Given that each population has specific food patterns, similar studies, especially prospective ones, are recommended in different age groups and populations.

## Data Availability

The datasets generated and/or analyzed during the current study are not publicly available, but are available from the corresponding author on reasonable request.

## Abbreviations

NAFLD: Non-alcoholic fatty liver disease
DASH: Dietary approaches to stop hypertension
OR: Odds ratio
BMI: Body mass index
FFQ: Food frequency questionnaire
ALT: Alanine aminotransferase
AST: Aspartate aminotransferase
GGT: Gamma-glutamyltransferase
FBG: Fasting blood glucose
LDL-c: Low-density lipoprotein cholesterol
HDL-c: High-density lipoprotein cholesterol
TC: Total cholesterol
TG: Triglycerides
WC: Waist circumference
HC: Hip circumference
IPAQ: International physical activity questionnaire
